# Diagnosing Heart Failure with Preserved Ejection Fraction in Obese Patients

**DOI:** 10.3390/jcm14061980

**Published:** 2025-03-14

**Authors:** Marino Basha, Evdoxia Stavropoulou, Anastasia Nikolaidou, Georgios Dividis, Emmanouela Peteinidou, Panagiotis Tsioufis, Nikolaos Kamperidis, Kyriakos Dimitriadis, Theodoros Karamitsos, George Giannakoulas, Konstantinos Tsioufis, Antonios Ziakas, Vasileios Kamperidis

**Affiliations:** 1 1st Department of Cardiology, AHEPA Hospital, School of Medicine, Aristotle University of Thessaloniki, 54124 Thessaloniki, Greece; emanouilmarinos@gmail.com (M.B.); evdostavrop@gmail.com (E.S.); natasa_nikol@hotmail.com (A.N.); gdvds.med@gmail.com (G.D.); emma2405@windowslive.com (E.P.); karamits@gmail.com (T.K.); g.giannakoulas@gmail.com (G.G.); tonyziakas@hotmail.com (A.Z.); 2 1st Department of Cardiology, Ippokrateion Hospital, School of Medicine, National and Kapodistrial University of Athens, 11528 Athens, Greece; ptsioufis@gmail.com (P.T.); dimitriadiskyr@yahoo.gr (K.D.); ktsioufis@gmail.com (K.T.); 3 Department of IBD, St. Mark’s Hospital, Imperial College London, London HA1 3UJ, UK; nkamperidis@yahoo.com

**Keywords:** obesity, heart failure, preserved ejection fraction, HFpEF, diastolic dysfunction, echocardiography, CMR, CCTA, BNP

## Abstract

Obesity is a current pandemic that sets all affected individuals at risk of heart failure (HF), and the majority of them will develop the clinical syndrome of HF with preserved ejection fraction (HFpEF). The diagnosis of HFpEF is challenging as it is based on the detection of subtle functional and structural remodeling of the heart that leads to diastolic dysfunction with increased left ventricular (LV) filling pressures and raised natriuretic peptides (NPs). The accurate diagnosis of HFpEF is even more challenging in patients who are obese, since the echocardiographic imaging quality may be suboptimal, the parameters for the evaluation of cardiac structure are indexed to the body surface area (BSA) and thus may underestimate the severity of the remodeling, and the NPs in patients who are obese have a lower normal threshold. Moreover, patients who are obese are prone to atrial fibrillation (AF) and pulmonary hypertension (PH), making the evaluation of diastolic dysfunction more strenuous. The current review aims to offer insights on the accurate diagnosis of HFpEF in patients who are obese in different clinical scenarios—patients who are obese in different clinical scenarios—such as in sinus rhythm, in atrial fibrillation, and in the case of pulmonary hypertension—by applying multimodality imaging and clinical diagnostic algorithms.

## 1. Introduction

Obesity has emerged as a global pandemic, affecting millions of patients across all age groups and geographic areas. Driven by sedentary lifestyles, unhealthy dietary patterns, and socioeconomic factors, its prevalence has skyrocketed. Almost 20–25% of the European population suffers from obesity, which increases the risk of chronic diseases like diabetes, hypertension, hyperlipidemia, atrial fibrillation, thromboembolism, atherosclerosis, and HF, straining healthcare systems worldwide [1,2].

It has been proven that obesity, through a number of pathophysiologic mechanisms, leads to LV diastolic dysfunction [3] and cardiac remodeling, which in turn may precipitate the development of HFpEF [4,5]. Cardiac fibrosis is a major deleterious effect of obesity that leads to HFpEF [6]. This occurs through the increased deposition of collagen in the extracellular matrix, which is driven by adipose tissue, making the myocardium stiffer and thus opposing the relaxation of the heart [7]. The proliferation of epicardial adipose tissue and its infiltration into the myocardium is excessive in patients who are obese and alters the geometry of the heart chambers, causing dilation and hypertrophy [8]. Moreover, the secretion of cytokines, such as activin A, connective tissue growth factor, insulin-like growth factor-1, growth differentiation factor-11, transforming growth factor-β, and metalloproteinases, by the epicardial adipose tissue has been shown to induce fibrosis [9,10]. Adipose tissue has also been shown to produce angiotensin-II and aldosterone that reach the myocardium and induce remodeling, which increases the LV wall stress [11,12]. The altered LV structure and diastolic dysfunction resulting from obesity lead to HFpEF.

The diagnosis of HFpEF may be challenging for clinicians, since the assessment of diastolic function according to the American Society of Echocardiography/European Association of Cardiovascular Imaging recommendations requires the assessment and measurement of several echocardiographic parameters [13]. As a result, there is significant underdiagnosis of this condition, especially among patients who are obese (defined as body mass index (BMI) > 30 kg/m^2^) [14,15]. The excess body fat of patients with obesity has a negative impact on the image quality of echocardiography [16] due to signal attenuation [17]. The interpretation of echocardiographic studies is also a point of debate, because adjustment for body size with the use of the BSA, the current standard, may lead to the misinterpretation of structural alterations in the hearts of patients who are obese [18]. Alternatively or additionally to echocardiography, the natriouretic peptides may be measured for the diagnosis of HFpEF. However, it has been shown that the NP levels are lower in patients with HFpEF who are obese compared to their non-obese counterparts, although both groups have higher levels than healthy individuals [19].

With HFpEF representing more than half of the cases of HF (56%) worldwide and with a continuous rise in incidence, a 37% relative increase over two decades [20,21], the need for an accurate and timely method of diagnosis is clear. The current literature review aims to enlighten the diagnosis of HFpEF in patients who are obese (Graphical Abstract).

## 2. HFpEF Diagnosis Based on Guidelines

The path towards a definitive diagnosis of HFpEF is nuanced and sometimes obscure. According to the European Society of Cardiology Guidelines, HF is a clinical syndrome consisting of cardiovascular symptoms and/or signs that arise due to underlying impairment of the structure and/or function of the heart [22]. However, based on the American College of Cardiology/American Heart Association Guidelines, apart from symptomatic HF, HF includes patients at risk of developing HF due to comorbidities (Stage A) and those with sub-clinical alterations in their cardiac structure and/or function (Stage B) who are asymptomatic [23]. Thus, the point of focus of clinical investigation, irrespective of the presence of symptoms, remains the identification of cardiac structural abnormalities and LV diastolic dysfunction by echocardiography. Two distinct algorithms have been proposed by the American Society of Echocardiography/European Association of Cardiovascular Imaging for the echocardiographic diagnosis of diastolic dysfunction. The selection of the appropriate algorithm is determined by the left ventricular ejection fraction (LVEF), which is determined to be either preserved or reduced, and the presence or absence of LV myocardial disease, defined as any structural LV remodeling or subtle LV endomyocardial dysfunction [13]. Hence, to start exploring the diagnosis of HFpEF, first the LVEF and then the type of LV remodeling and subclinical dysfuntion have to be evaluated in order to define which algorithm to apply for the assessment of LV diastolic function: the one for preserved LVEF and normal cardiac structure or the second one for reduced LVEF or preserved LVEF with cardiac structural remodeling or functional impairment.

## 3. Left Ventricular Structure and Systolic Function Evaluation in Patients Who Are Obese

**Figure 1:**
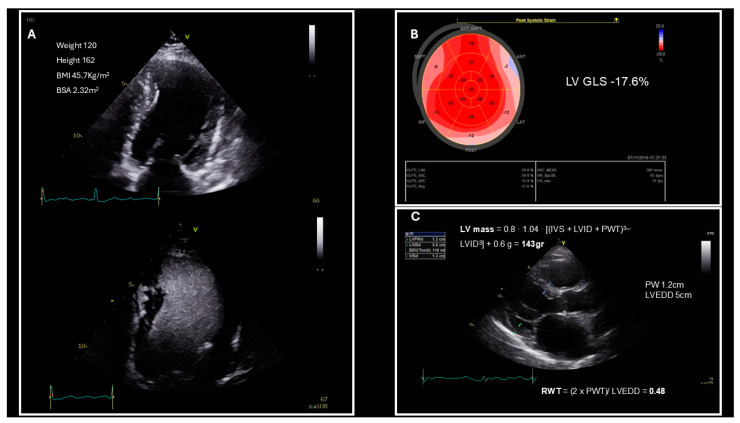
Challenges in evaluating left ventricular (LV) function and structure in the obese; (**A**) for better endocardial delineation IV contrast is used to measure LV ejection fraction accurately. (**B**) LV global longitudinal strain (GLS) is assessed to detect subtle endomyocardial LV dysfunction. (**C**) LV relative wall thickness and mass are estimated based on the LV wall thickness and end-diastolic diameter. In the case of this female obese patient, LV mass index (LVMI) calculated with body surface area (BSA) indexing falls into the normal category (61 g/m^2^ < 95 g/m^2^), while for another female of the same height, but with a normal body mass index (BMI) (height 162 cm, weight 49 kg, BMI = 18.7 kg/m^2^ and BSA = 1.48 m^2^), the LVMI (97 g/m^2^) along with the relative wall thickness (RWT) = 0.48 would indicate LV concentric hypertrophy. Abbreviations: LVEDD, left ventricular end-diastolic diameter; PW, posterior wall.

The impact of obesity on patients’ cardiac structure and function is a prominent topic of research, and its association with negative impacts on these aspects is well established according to the current literature. LV hypertrophy is a common finding in patients who are obese, with 56% of them displaying findings that are indicative of this feature [24], which is captured on echocardiography as a substantial increase in LV mass and relative wall thickness (RWT) [25,26]. The LV mass is increased not only in the setting of obesity, but also in patients with obesity-associated comorbidities, such as hypertension and diabetes, which are independently linked to increased LV mass values [27]. Even though the BMI does not have a direct impact on the LVEF, functional assessment of the heart in patients who are obese with a preserved LVEF should include the evaluation of the LV global longitudinal strain (Figure 1B), as it is considered to be an early indicator of the intramyocardial impairment that leads to subclinical systolic dysfunction and an independent predictor of diastolic dysfunction in patients who are obese [28]. The correlation between patients’ BMI and their LV global longitudinal strain is well-established, with the current literature indicating a decrease in the latter as the BMI increases [27,29,30]. Moreover, in HFpEF patients both longitudinal and circumferential strain values are impaired as opposed to their counterparts in the control group and exhibit an inverse correlation with LVEF [31].

According to the aforementioned literature, patients who are obese have a high likelihood of presenting with structural and functional myocardial alterations. Nevertheless, it is imperative that the physician evaluates the LVEF and the parameters that indicate LV remodeling in order to select the appropriate algorithm for diastolic dysfunction evaluation. This does not come without the difficulties imposed by obesity (Table 1), as echocardiographic studies are less likely to be conclusive in patients who are obese due to excess adipose tissue, both visceral and subcutaneous, diminishing the amount of ultrasound waves returning to the probe and simultaneously increasing the chest wall thickness, which limits wave penetration. This results in poor image quality and thus increased difficulty in demarcating endocardial borders and in assessing the dimensions of the heart chambers [16,17]. To address this challenge, clinicians can follow some actionable steps to enhance the quality of echocardiographic images obtained for patients who are obese. Using lower-frequency transducers is crucial for increasing tissue penetration, a key requirement in evaluating patients with obesity. Nevertheless, this comes at the cost of a reduced image resolution, which can be improved by reducing the image depth and width to increase the frame rate, adjusting the focus point based on the structure being examined, and appropriately increasing the grayscale gain and dynamic range.

Alternatively, the use of ultrasound-enhancing agents is recommended (Figure 1A), as it raises the accuracy of LVEF estimation to the same level as that in patients who are not obese [16]. In cases of poor acoustic windows and the echocardiographic image quality remaining suboptimal despite the use of contrast-enhanced echocardiography, alternative imaging modalities may be employed.

Cardiac magnetic resonance (CMR) excels as an alternative or complementary imaging modality in cases of poor acoustic windows and concomitantly provides an insight into the aetiology of HFpEF. CMR, being the gold standard for defining cardiac geometry, provides a precise assessment of the LA and LV volumes, mass, and function by the LVEF. Additionally, it provides the unique ability of tissue characterization, thereby allowing physicians to quantify local fibrosis via late gadolinium enhancement and diffuse fibrosis through T1 mapping [32]. An expansion of extracellular space, attributed to increased interstitial fibrosis, is observed in many HFpEF patients and is directly correlated to the extracellular volume [33]. Several studies have shown the promising role of the CMR-derived extracellular volume in the evaluation of patients with HFpEF, as higher levels of fibrosis have been associated with LV chamber stiffness and adverse outcomes in patients with HFpEF [34,35]. Furthermore, the myocardial deformation, assessed using strain analysis, can be obtained by processing CMR cine sequences that are included in standard acquisition protocols [32]. The CMR-derived GLS has shown promising prognostic values in various cardiac conditions [36], while the LA strain, obtained by CMR, was shown to have an incremental prognostic value in patients with a preserved LVEF [37].

Cardiac computed tomographic angiography (CCTA) is mainly used in everyday clinical practice to uncover and evaluate coronary artery disease; however, due to its high spatial resolution, it is feasible to measure the LV volumes at the end-diastolic and end-systolic phases and the LVEF and LV mass using the same three-dimentional data set without the need for additional contrast or radiation exposure. The contrast opacification of the LV is excellent during cardiac CCTA and facilitates delineation of the endocardial border from the contrast-filled LV cavity [38]. Moreover, studies have shown good correlations between LA volume assessment using cardiac CT and that using echocardiography [39,40]. Wang et al. showed that CCTA could also be useful in analyzing the LV strain in HF patients, with CCTA-derived three-dimensional-GLS being reliable for quantitatively assessing myocardial mechanical changes in HF patients [41], while studies from other patient populations support that CCTA-acquired LA strain measurements are accurate and highly reproducible [42,43,44]. However, contraindications such as contrast allergies and renal insufficiency preclude the use of CCTA.

Another challenge in patients who are obese is the reliable evaluation of their LV remodeling, which requires the measurement of the LV mass index (LVMI) to the BSA and the RWT (Figure 1C) [45]. Concentric remodeling and concentric LV hypertrophy are common findings in patients with HFpEF and serve an integral role in establishing its diagnosis. A normal LVMI accompanied by an elevated RWT (>0.42) is indicative of concentric remodeling, while an increase in both the LVMI (>95 g/m^2^ in women, >115 g/m^2^ in men) and RWT is suggestive of concentric LV hypetrophy [45]. In the setting of obesity, the LVMI values may be inappropriately deflated due to an elevated BSA, which in turn may lead to the underestimation of myocardial remodeling, especially in cases where the patient’s weight may fluctuate [16]. Indexing the LV mass to the height of the patient has been proposed to more accurately assess LV remodeling in the case of obesity, with an LVMI > 47 g/m^2.7^ in women and >50 g/m^2.7^ in men unveiling LV hypertrophy in those with a BMI ≥ 30 kg/m^2^ [18]. Accurate assessment of the hypertrophic type of LV remodeling is of paramount importance, as it is linked with worse patient outcomes [46].

## 4. Diastolic Dysfunction Assessment in Individuals Who Are Obese in Sinus Rhythm

After the LVEF and the LV remodeling have been adequately assessed, the next step towards the diagnosis of HFpEF is to explore the LV diastolic function. The diastolic dysfunction is assessed using several echocardiographic parameters (Table 2), including the maximum velocity of passive mitral filling (E), the maximum velocity of active mitral filling (A), the early diastolic mitral annulus velocity (e′), and the E/A and E/e′ ratios. Other key parameters include the maximum tricuspid regurgitation velocity (TR Vmax), the systolic pulmonary artery pressure, and the left atrial volume index (LAVI). Since obesity is linked to LV diastolic dysfunction [26,47], these indices are more likely to show changes that are suggestive of diastolic dysfunction in individuals with a raised BMI compared to those with a normal BMI [48,49].

**Figure 2:**
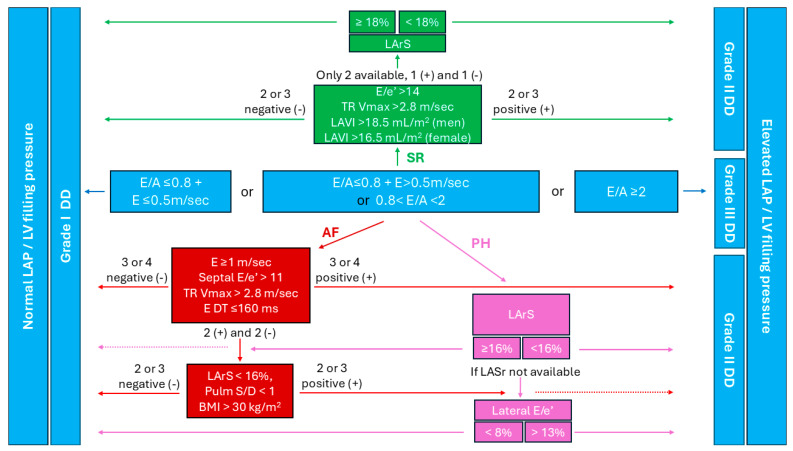
Algorithm for diastolic dysfunction assessment in patients who are obese with body mass index (ΒΜΙ) >30 kg/m^2^. After evaluating the blue boxes, follow the green in case of sinus rhythm (SR), the red for atrial fibrillation (AF), and the purple for pulmonary hypertension (PH). Abbreviations: E DT, mitral E wave deceleration time; DD, diastolic dysfunction; LAP, left atrial pressure; LArS, left atrium reservoir strain; LAVI, left atrium volume index to height; LV, left ventricular; Pulm S/D, pulmonary vein systolic/diastolic velocity ratio; TR Vmax, tricuspid regurgitation maximum velocity.

The American Society of Echocardiography/European Association of Cardiovascular Imaging guidelines provide two structured algorithms for the assessment of diastolic dysfunction, as explained previously [13]. In patients who are obese, the LVEF is usually preserved, while the LV is remodeled. Therefore, it is more appropriate to follow the algorithm for a preserved LVEF with cardiac abnormalities, which means that the initial patient evaluation includes the measurement of the E/A ratio and E wave (Figure 2). If the E/A ≤ 0.8 and the E > 0.5 m/sec, or 0.8 < E/A < 2, then assessment of the E/e′ ratio, LAVI, and TR Vmax must follow [13]. Obesity is associated with a higher likelihood of diastolic dysfunction, but it also complicates the accuracy of measuring and interpreting these parameters.

**Figure 3:**
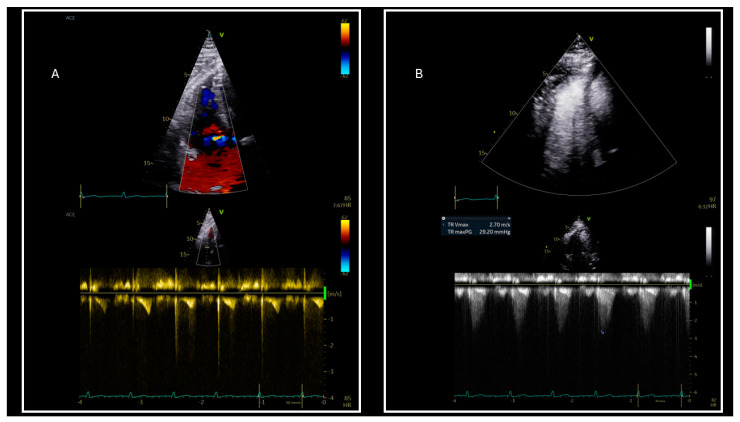
Improvement in accuracy of tricuspid regurgitation (TR) maximum velocity (Vmax) assessment with the use of intravenous contrast enhancing agent. The continuous wave Doppler evaluation of TR Vmax (**A**) without contrast is suboptimal. (**B**) TR Vmax with contrast is clearly detected.

Signal attenuation and poor acoustic windows due to excessive bodyfat have a negative impact on image clarity [16,17], thereby making precise TR Vmax measurement a challenge. The use of contrast-enhanced echocardiography has been proven to be highly effective in overcoming this (Figure 3), as agitated saline, sonicated albumin microspheres, and saline contrast have been shown to improve the TR Vmax estimation accuracy by optimizing the TR spectra quality in 73–100% of cases [50,51].

Underestimation of the LA size is a common pitfall of indexing to the BSA in patients who are obese. This occurs due to the unaccounted-for BSA increase with increases in the BMI and the subsequent deflation of LAVI measurements [16,18]. To address this, indexing factors that are unrelated to weight, such as height, are being proposed as potential substitutes to the BSA for normalizing heart parameters. These methods expand the pool of patients who are obese who meet the criteria for LA dilation according to LAVI, reducing type 2 errors [16,18]. For patients with a BMI < 30 kg/m^2^, indexing to the BSA does not affect the reliability of the results. In patients who are obese, however, the choice of indexing methods should be personalized for each case. If the LA is found to be dilated (>34 mL/m^2^) with BSA indexing, then the physician can proceed to grade the diastolic dysfunction based on the guidelines but cannot accurately describe the severity of the LA enlargement and should therefore mention that the LAVI may be underestimated. If, on the other hand, the LAVI is ≤ 34 mL/m^2^, then the physician should either use height-based indexing, with the cut-off set at 18.5 mL/m^2^ for men and 16.5 mL/m^2^ for women, or the anteroposterior linear dimension obtained from the parasternal long-axis view, with the cut-off set at 4.1 cm for men and 3.9 cm for women. BSA indexing can also be used in the obese, but the cut-off for normal values should be reduced from 34 mL/m^2^, used for patients with a BMI < 30 kg/m^2^, to 29 mL/m^2^ for patients with a BMI > 30 kg/m^2^ (Table 3), while the ranges of 29–33 mL/m^2^, 34–39 mL/m^2^, and >40 mL/m^2^ are reserved for mild, moderate, and severe dilation, respectively [18].

In the case of only two of the three parameters for determining an increased LV filling pressure (E/e′, TR Vmax and LAVI) being available to the physician, with one being positive and the other negative, the results are deemed inconclusive [13]. However, the 2024 recommendations suggest that the LA reservoir strain should be used to assist in making a definitive diagnosis of elevated LV filling pressure in these cases. Specifically, a measurement of the LA reservoir strain ≥ 18% would indicate normal LA pressure, while an LA strain < 18% would indicate elevated LA pressure [52]. This is significant when evaluating LV diastolic dysfunction in patients who are obese, since an increased BMI is linked to LA strain impairment during all phases of LA function [29,53,54]. The LA reservoir strain demonstrates stepwise impairment, progressively worsening from patients with normal BMI to those who are overweight, and finally to those who are obese, while the contraction and conduit phase LA strains are affected to the same extent by the patient being overweight and the patient being obese [54]. In addition to this, the LA reservoir strain has been shown to improve in patients who were previously obese who lost weight [53]. Regarding the effects of a raised BMI on the LA contraction strain specifically, the findings are not conclusive, as Chirinos et al. [55] found improved values in the obese group in their study.

Finding the LA pressure to be within the normal range in a symptomatic patient should raise suspicion of coronary artery disease, since the symptoms cannot be attributed to HFpEF. In such cases, CCTA follows as the next step in the diagnostic algorithm [13]. Performing CCTA in patients who are obese presents its own set of challenges, as the image quality may be compromised due to signal attenuation [56], necessitating high dosages of radiation and contrast material and therefore increasing the risk of contrast-associated nephropathy [57]. Additionally, limitations regarding equipment dimensions and patient habitus can further complicate the procedure [58].

## 5. Diastolic Dysfunction Assessment in Individuals Who Are Obese in Atrial Fibrillation

AF is the cardiac arrythmia with the highest prevalence in the general population, and its prevalence is growing exponentially with no signs of slowing down [59]. There is a clear association between AF and obesity, with each one-point increase in BMI increasing the risk of AF by 4% [60]. Obesity-induced LA dilation seems to be the main factor that precipitates AF in these patients [60,61]. Nevertheless, the pathophysiologic mechanisms linking obesity to AF extend far beyond LA enlargement and include epicardial adipose tissue that infiltrates the myocardium, causing inflammation, oxidative stress, and fibrosis through the secretion of adipokines [9,10,62]; neurohormonal stimulation due to overactivation of the RAAS pathway that induces myocardial remodeling [63,64]; and obesity-associated comorbidities such as hypertension [65], CAD, diabetes, sleep apnea, and LV diastolic dysfunction [66].

The evaluation of diastolic dysfunction in the setting of AF is particularly challenging due to the irregularity of atrial activity and its dilation, irrespective of the filling pressures [13]. No clear-cut algorithm exists for such cases, although Nagueh et al. have proposed several markers of increased filling pressures: a mitral deceleration time ≤ 160 msec, a peak acceleration rate of mitral E velocity ≥ 1900 cm/sec^2^, an isovolumic relaxation time ≤ 65 msec, a deceleration time of pulmonary venous diastolic velocity ≤ 220 msec, an E/mitral propagation velocity ratio ≥ 1.4, and an E/e′ ratio ≥ 11 [13]. It is imperative to average the measurements in order to minimize the deviation caused by irregular LA contractility and obtain accurate results; this is either done over 10 consecutive cardiac cycles or 3 non-consecutive beats with RR intervals within 10–20% of the average [13].

**Figure 4:**
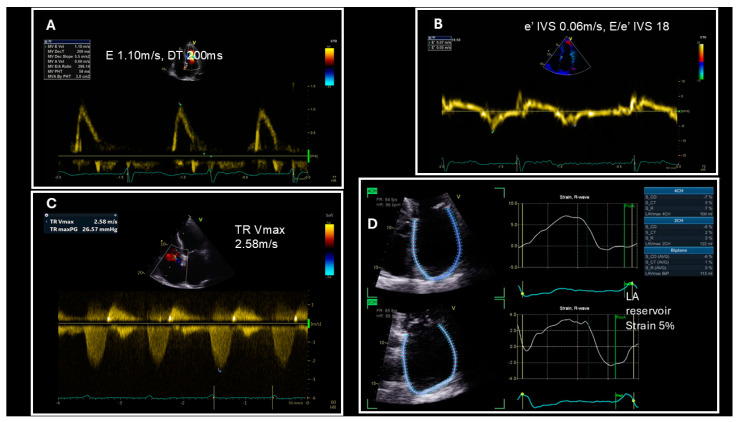
Echocardiographic assessment of left ventricular diastolic dysfunction in an obese patient with atrial fibrillation. (**A**) E wave velocity is >1 but deceleration time (DT) is >160 ms. (**B**) E/e’ ratio at the interventricular septum (IVS) is >11. (**C**) Tricuspid regurgitation (TR) Vmax is <2.8 m/s. Two parameters are positive and two are negative according to the diastolic dysfunction algorithm. (**D**) Left atrial (LA) reservoir strain is <16%. Hence, since the patient is obese (body mass index > 30 kg/m^2^), the LV filling pressure is elevated.

Although none of the aforementioned parameters have a strong enough correlation to the LV filling pressure to be used as a standalone test, the 2024 recommendations by Smiseth et al. put forward a diagnostic algorithm that simultaneously evaluates multiple of these measurements along with the patient’s BMI and gives an accurate estimation in 75% of cases [52]. The criteria examined by the algorithm include a septal E/e′ ratio > 11, a TR Vmax > 2.8 m/sec, a mitral E velocity ≥ 1 m/sec, and a mitral deceleration time ≤ 160 msec (Figure 4). If three or more of these criteria are fulfilled, then a diagnosis of increased LV filling pressure can be made. On the other hand, if three or more of these criteria are negative, then the LV filling pressure is considered normal. Indeterminate cases, where the criteria are equally split, require further testing. The subset of criteria used in ambiguous cases include an LA reservoir strain of <16%, a pulmonary vein systolic/diastolic velocity ratio <1, and a BMI > 30 Kg/m^2^. If two or more of these are positive then elevated LV filling pressure is confirmed, while in the case that two or more are negative, the pressure is considered normal [52]. Obesity is the only non-echocardiographic parameter utilized in the algorithm, due to the BMI showing a moderate correlation (r = 0.25 with *p* < 0.01) to the LV filling pressure [67].

## 6. Pulmonary Hypertension and Diastolic Dysfunction in Obese

PH is another factor that perplexes the evaluation of diastolic dysfunction. Its pathogenesis is highly associated with obesity and obesity-related comorbidities [68]. Obstructive sleep apnea, which is present in up to 45% of individuals who are obese [69], precipitates hypoxia-mediated constriction and remodeling of the pulmonary vasculature. It does so through multiple nocturnal hypoxic episodes that cause the retention of CO_2_ and subsequent respiratory acidosis, a heightened sympathetic tone, pronounced fluctuations in intrapulmonary pressures, and a diminished capacity of the endothelium to mediate arteriolar dilation [70]. Obesity hypoventilation syndrome, present in 31% of patients with a BMI > 35 kg/m^2^, leads to hypoventilation due to an inability to fully expand the thoracic wall, while also causing obstruction of the upper airways and abnormally large negative pressures during inspiration. This condition is a major cause of PH in the obese as its effects on respiration are exerted throughout the day, unlike obstructive sleep apnea [70]. The elevated LV filling pressures that are commonly found in patients who are obese with HF can be transmitted retrogradely to the LA, then to the pulmonary vessels, and, then, finally, to the right heart chambers. This chronicity of high pulmonary pressures, secondary to diastolic dysfunction in the obese, leads to the remodeling of lung vessels [70]. An indirect way for individuals who are obese to develop PH is through the use of appetite reducing medications, such as fenfluramine and dexfenfluramine, for which the primary intention is weigh reduction [70,71]. At last, the high probability of venous thrombi formation in patients who are obese is suggested to increase the likelihood of pulmonary embolism, a condition that abruptly and permanently increases pressures in the lung vasculature and may lead to chronic thromboembolic PH [70]. Other mechanisms through which obesity and its related comorbidities lead to PH are being investigated, as these conditions have been shown to impair blood vessel function, increase oxidative stress, and disrupt the balance between endothelin and nitric oxide [70].

Obesity-related PH falls into two categories: one where the resistance of the pulmonary arteries is excessive (pre-capillary PH, i.e., group 1, group 3, and group 4 PH) and another where left heart disease, namely diastolic dysfunction, causes a rise in the LV filling pressures that is transmitted retrogradely to the pulmonary vessels (post-capillary PH, i.e., group 2 PH). The latter is the most common cause of PH, since 65–80% of cases are attributed to left heart disease [52,72].

Due to the difference in treatment modalities between the four groups of PH, the need to distinguish between them is obvious. It is of paramount importance to differentiate between the pre- and post-capillary PH, since the latter affects the majority of PH patients and implies that the left heart disease should be treated. This is achieved with high accuracy using right heart catheterization for the evaluation of the mean pulmonary artery pressure and the LA pressure, measured as pulmonary capillary wedge pressure. Since this is an invasive method and access to it may be limited, a non-invasive alternative which relies on echocardiographic measurements has been proposed. This method is conducted to evaluate the LV filling pressure in all patients with suspected PH before the use of right heart catheterization, due to the chances being in favor of post-capillary PH [52].

Since post-capillary PH is characterized by elevated LV filling pressures, while pre-capillary PH is associated with normal LV filling pressures, the proposed algorithm for evaluating LV diastolic dysfunction differentiates between pre- and post-capillary PH. Accordingly, the mitral E/A ratio, LA reservoir strain, and lateral E/e′ ratio are assessed. The process begins with an assessment of the mitral E/A ratio. Values ≤ 0.8 indicate a lack of LV diastolic dysfunction, whereas values ≥ 2 confirm its existence. When the E/A ratio lies between 0.8 and 2, the result is deemed inconclusive and LA reservoir strain measurement is recommended, with values ≥ 16% suggesting normal LV filling pressures, while values < 16% point to elevated LV filling pressures. If LA reservoir strain measurement is unavailable, the lateral E/e′ ratio can be utilized. The LA reservoir strain has an accuracy of 85%, while the lateral E/e′ ratio demonstrates an accuracy of 86% in assessing LV diastolic dysfunction. A lateral E/e′ ratio < 8 indicates normal LV filling pressures, while a ratio > 13 confirms increased LV filling pressures. However, when the lateral E/e′ ratio is between 8 and 13, it does not provide a definitive assessment. This stepwise approach helps to distinguish between pre- and post-capillary PH while addressing the limitations of each diagnostic parameter [52].

## 7. Applying Clinical Algorithms in Patients Who Are Obese for HFpEF Diagnosis

Two clinical algorithms have been suggested to aid in the diagnosis of patients with suspected HFpEF, the HFA-PEFF (Heart Failure Association Pretest Probability of Heart Failure with Preserved Ejection Fraction) [45] and the H_2_FPEF (Heavy, Hypertension, Atrial Fibrillation, Pulmonary Hypertension, Elder, and Filling Pressures) scores [73], both of which quantify the risk of HFpEF with the use of a numeric score [22,23] (Table 4).

### 7.1. H_2_FPEF Diagnostic Algorithm

The H_2_FPEF algorithm includes six parameters that are scored according to their significance in predicting HFpEF. These are AF, obesity, an age > 60 years, treatment with two or more antihypertensives, an echocardiographic E/e’ ratio > 9, and an echocardiographic pulmonary artery systolic pressure > 35 mmHg. Patients that are positive for AF are assigned three points, obesity is scored as two points, and all other parameters are given one point each. This creates a scoring range of 0–9 in the H_2_FPEF diagnostic algorithm, with the likelihood of HFpEF doubling for every additional point [73,74]. This algorithm highlights the connection of obesity with HFpEF, since it is scored with two points, while all the other parameters apart from AF are scored with one point each. Of note is that obesity is not merely one of the score’s parameters, but may precipitate almost all of the other parameters of the score such as AF, hypertension, PH, and an elevated LV filling pressure.

### 7.2. HFA-PEFF Diagnostic Algorithm

The HFA-PEFF diagnostic algorithm follows four steps to evaluate HFpEF.

The initial step (P) involves assessing signs and symptoms consistent with HF, identifying comorbidities or risk factors like obesity, and conducting simple tests to rule out other conditions, cardiac and non-cardiac, that may have a clinical presentation similar to HFpEF. Obesity is a disease that may provoke symptoms similar to HF and, concomitantly, it may be the ground pathophysiology leading to it. Thus, the initial work-up is not adequate in the obese and the next step (E), that of diagnostic assessment, should be activated to confirm the diagnosis [45].

The second step (E) focuses on comprehensive echocardiography and NP scoring. Based on these, a scoring system evaluates the cardiac structural and functional alterations that can be seen through echocardiography, as well as the circulating NP levels. Each category includes cut-off values that define major and minor criteria. Meeting one or more major criteria in a category earns two points, while meeting one or more minor criteria earns one point. This means that the HFA-PEFF score ranges from 0 to 6, with a score of 5 or greater confirming the diagnosis of HFpEF and a score of 1 or less making it highly unlikely [45]. Echocardiographic functional assessment is conducted using the e’ wave, E/e’ ratio, TR Vmax, and LV global longitudinal strain. The major functional criteria include a septal e′ < 7 cm/sec or lateral e′ < 10 cm/sec, a mean septal and lateral E/e′ ratio > 15, and a TR Vmax > 2.8 m/sec or systolic pulmonary artery pressure > 35 mmHg. Minor functional criteria include a mean septal and lateral E/e′ ratio in the 9–14 range and an LV global longitudinal strain < 16% [45]. The structural assessment involves LAVI, LVMI, and LV-RWT measurements. The major structural criteria include a LAVI > 34 mL/m^2^ for patients who have sinus rhythm or a LAVI > 40 mL/m^2^ for those with AF, and a LVMI > 149 g/m^2^ in men or LVMI > 122 g/m^2^ by an RWT > 0.42 in women accompanied. The minor structural criteria include a LAVI in the range of 29–34 mL/m^2^ for sinus rhythm or 34–40 mL/m^2^ for those with AF, an LVMI > 115 g/m^2^ in men or an LVMI > 95 g/m^2^ in women, an RWT > 0.42, and LV wall thickness in the end of the diastole ≥ 12 mm [45]. Indexing of the LV mass and LA volume were performed using the BSA; therefore, the cut-off values may be inappropriately high for patients who are obese. In this subset of patients, it may be more appropriate to index the LA volume to the height^2^ and the LV mass to the height^2.7^ according to the European Society of Cardiology 2018 Guidelines on Hypertension Management [75]. These guidelines set the cut-off values for a normal LAVI at ≤18.5 mL/m^2^ for men and ≤16.5 mL/m^2^ for women, while the threshold for a normal LVMI is set at ≤50 g/m^2.7^ for men and ≤47 g/m^2.7^ for women [18,75].

B-type natriuretic peptide (BNP) and the N-terminal fragment of proBNP (NT-proBNP) are the NPs with the longest half-lives and are indicative of increased myocardial wall stress. Their values are increased in the settings of both HFpEF and HF with reduced EF (HFrEF) [76,77], with their levels being higher in the latter but with no cut-off value existing to differentiate between the two [77]. NPs are part of the definition of HFpEF according to the European Society of Cardiology Guidelines; symptoms or signs of HF, a preserved LVEF, and elevated NPs define HFpEF even without echocardiography confirmation [22]. In the HFA–PEFF algorithm, evaluation of the BNP and NT-proBNP levels is of great significance for scoring the risk of HFpEF, as it is one of the three categories that is evaluated. Regarding patients with sinus rhythm, an NT-proBNP > 220 pg/mL and a BNP > 80 pg/mL are deemed major criteria, while an NT-proBNP 125–220 pg/mL and a BNP 35–80 pg/mL are considered minor [45]. However, before applying these cut-off values, many cofounders that alter the amount of circulating NPs have to be considered: their levels may be decreased due to obesity and a number of other factors such as insulin resistance [78] and increased GFR [19,79,80], or may be increased due to age, hypertension [81], atrial fibrillation, chronic kidney disease, and female gender [82]. Accordingly, for patients with AF, higher cut-off values have been applied as diagnostic of HFpEF: NT-proBNP > 660 pg/mL and BNP > 240 pg/mL are major criteria, while NT-proBNP 375–660 pg/mL and BNP 105–240 pg/mL are minor. Subsequently, for patients who are obese, significantly lower cut-off values have to be set considering that the alterations in the baseline NP levels seen in individuals who are obese may be up to three times lower than those with a normal BMI. Therefore, Madamanchi et al. suggested that a BNP of 54 pg/mL may be set as the new threshold (Table 3) for ruling out HFpEF in patients who are obese [79].

Apart from the NPs, other biomarkers may have a role in pointing towards the diagnosis of HFpEF in patients who are obese. High-sensitive cardiac troponin T, a biomarker of subclinical myocardial injury, displays a linear increase with patients’ BMI, and is associated with the development of incident HF [83]. Galectin-3, a biomarker of cardiac inflammation and fibrosis, is increased in patients who are obese, and elevated levels of galectin-3 along with higher BMI levels are linked to an increased risk of future HF [84]. However, these biomarkers are not a part of the HFA-PEFF score.

The third step (F1) is activated if the HFA–PEFF score is inconclusive (2–4 points). Then, diastolic stress testing is recommended in order to definitively answer the question of whether patient’s symptoms are secondary to HFpEF. The methods can either be non-invasive or invasive, with the latter providing more reliable results [45]. In clinical practice, =the exercise stress echocardiography test is usually used to shed light on the conundrum of diastolic dysfunction; however, patients who are obese have increased chances of poor image quality or an inconclusive test due to poor exercise tolerability.

The final step (F2), etiological investigation, incorporating advanced imaging techniques as well as genetic testing, biopsies, and specialized laboratory tests, such as serum electrophoresis, serum free light chains, serum angiotensin-converting enzyme, plasma metanephrines, has to be applied to identify the underlying cause [45] in patients who are obese when a specific etiology of heart failure is suspected, despite the difficulties described and the possibility that obesity itself may be the underlying etiology.

### 7.3. Accuracy and Limitations of the Diagnostic Algorithms

**Figure 5:**
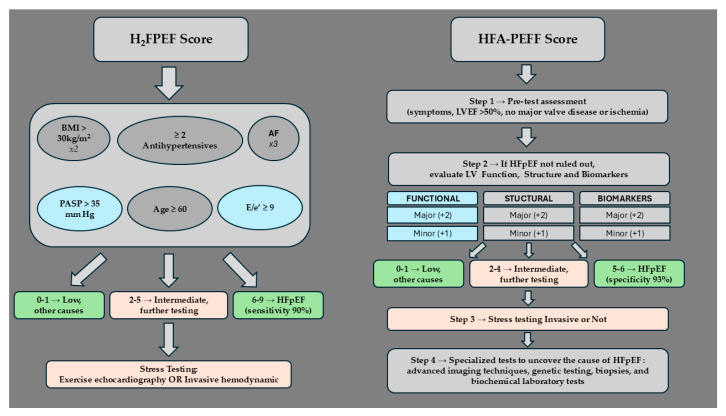
Stepwise application of the two diagnostic algorithms for HFpEF diagnosis. The corresponding color denotes comparable steps between the two algorithms. Abbreviations: AF, atrial fibrillation; BMI, body mass index; H2FPEF, Heavy, Hypertension, Atrial Fibrillation, Pulmonary Hypertension, Elder, and Filling Pressures; HFA-PEFF, Heart Failure Association Pretest Probability of Heart Failure with Preserved Ejection Fraction; HFpEF, heart failure with preserved ejection fraction.

Both algorithms have been evaluated in small cohorts and validated for use in the general population [85]. The H2FPEF score was developed using invasive hemodynamic testing as the gold standard and had good discriminatory performance with an area under the operating curve of 0.88. A H2FPEF score of >2 had a sensitivity of 89–90% in detecting HFpEF and a H2FPEF score < 6 had a specificity of 82% in ruling out HFpEF [86]. In contrast, the HFA-PEFF algorithm was an expert consensus recommendation, with diagnostic accuracy of 90% as determined by the area under the curve. A high HFA-PEFF score (5–6 points) could diagnose HFpEF with a high specificity of 93% and a low HFA-PEFF score (0–1 points) could rule out HFpEF with a sensitivity of 99% [87] (Figure 5).

While both algorithms have shown promise in diagnosing HFpEF, the concordance between them is reported to be low, suggesting that they may not be interchangeable and highlighting the ongoing challenge of accurately diagnosing patients with HFpEF [88]. When applying these algorithms in patients who are obese, specific challenges and limitations apply. In the H2FPEF score, obesity is allocated two points, increasing the possibility of HFpEF diagnosis in patients who are obese. On the other hand, the HFA-PEFF score takes into account the NT-proBNP levels; however, obesity is associated with low circulating natriuretic peptide levels, which may lead to under-diagnosing HFpEF in individuals who are obese. Many patients who are obese with HFpEF exhibit normal or marginally elevated LV filling pressures at rest and elevated LV filling pressures only during exercise. Thus, performing additional exercise echocardiography or invasive catheterization may be mandatory in highly suspicious cases [89]. In a prospective study with 78 patients with obesity and dyspnea, at least one third had clinically unrecognized HFpEF which was uncovered upon invasive cardiopulmonary exercise testing. Both patients with and without HFpEF had similar biomarkers, resting echocardiography findings, and HFpEF risk scores before invasive evaluation [14]. These findings suggest a considerable under-recognition of HFpEF among individuals who are obese and highlight the limitations of non-invasive diagnostic tools in the identification of HFpEF in the setting of obesity. Clinicians should be aware of the limitations of each algorithm when applying them in patients who are obese in order to arrive at an accurate diagnosis.

## 8. Advancing HFpEF Diagnosis in Patients Who Are Obese: Key Areas for Future Research

### 8.1. Multimodality Assessment in Patients Who Are Obese

Transthoracic echocardiography plays a pivotal role in the definition of HFpEF. Due to its low cost, vast availability, and use of Doppler-derived measurements, it is the initial test recommended for the evaluation of patients who are obese with possible HFpEF. CCTA and CMR both play a key role in assessing cardiac structure and function, especially in patients for whom suboptimal echocardiographic images are obtained, and are capable of providing important diagnostic and prognostic information. Especially, the use of advanced cardiac imaging techniques that incorporate strain analysis is a promising field that requires additional studies in order to better clarify the role of CCTA- and CMR-derived strain in the assessment of patients with HFpEF. A potential approach in patients who are obese with HFpEF would be the use of multimodality imaging, combining imaging data from echocardiography and cardiac CT or MRI, along with clinical and biomarker findings.

### 8.2. Gender Differences in Patients Who Are Obese

In addition to this, significant differences according to gender apply in HFpEF. The prevalence of HFpEF has been demonstrated to be higher in women than men, as women more commonly present traditional risk factors, such as obesity, diabetes, and hypertension, which contribute to the development of HFpEF, but also female-specific risk factors, such as sex hormones and menopause, which play a central role in the predominance of HFpEF in women [90]. Beyond differences in risk factors, there are also significant differences in outcomes according to gender, with women reporting a lower quality of life but overall better survival than men [91]. Women also have more severe diastolic dysfunction with higher left ventricular filling pressures compared to men [92]. The American Society of Echocardiography and the European Association of Cardiovascular Imaging recommend using gender-specific cut-off values for LV volumes, the LVEF, and the LV mass [93]. These reference values should be used according to gender and, when the LV volumes and mass are indexed to the BSA in patients who are obese, the same limitations mentioned before apply for both genders.

Thus, future research should focus on developing more precise and personalized diagnostic algorithms and investigating alternative methods for the diagnosis and stratification of patients who are obese with suspected HFpEF.

## 9. Conclusions

Individuals who are obese face a documented risk of developing HFpEF during their lifetime. The comprehensive echocardiographic assessment of the LV structure and function, along with the evaluation of diastolic dysfunction, incorporating the LA strain, remains the cornerstone in the diagnosis of HFpEF, although it is technically more challenging in the obese. The structural parameters the LVMI and LAVI are inappropriately underestimated in patients who are obese due to BSA indexing. To address this limitation, indexing to height has been proposed. Similarly, the current state of NP level evaluation in patients with a high BMI leads to underdiagnosis, as they exhibit significantly lower measurements. Reducing the cut-off value for HFpEF assessment in the obese has been suggested. Further studies have to be conducted in patients who are obese, taking into consideration their gender, to define robust cut-off values for the LVMI and LAVI that are indexed to height and NP levels that are indicative of HFpEF.

## Figures and Tables

**Table 1:** Diagnostic challenges in patients who are obese.

Poor image quality
Use of contrast agents for accurate left ventricular ejection fraction estimation
Use of contrast agents for accurate tricuspid regurgitation maximum velocity estimation
Underestimation of left ventricular mass index
Underestimation of left atrial volume index
Lower natriuretic peptides levels

**Table 2:** Echocardiographic parameters for assessing HFpEF.

**Left ventricular structure**	Left ventricular mass index
	Relative wall thickness
**Left ventricular systolic function**	Left ventricular ejection fraction
	Global longitudinal Strain
**Left ventricular diastolic function**	E wave velocity
	A wave velocity
	E/A ratio
	E/e’ ratio
	Tricuspid regurgitation Vmax
	Left atrial volume index
	Left atrial reservoir strain
	E wave deceleration time (in AF)
	Pulmonary vein Systolic/Diastolic velocity ratio (in AF)

AF, atrial fibrillation.

**Table 3:** Differences in cut-off values between lean versus patients who are obese.

	Lean	Obese
**Left atrial volume index—LAVI**	34 mL/m^2^, index to BSA	29 mL/m^2^, index to BSA
**Left ventricular mass index—LVMI**	95 g/m^2^ (women) 115 g/m^2^ (men), index to BSA	47 g/m^2.7^ (women) 50 g/m^2.7^ (men), index to height
**B-type Natriuretic peptide—BNP**	80 pg/mL	54 pg/mL

**Table 4:** Scoring of the H_2_FPEF and HFA-PEFF algorithms.

**H_2_FPEF Algorithm**				
**Parameters**				Score
**BMI > 30 kg/m^2^ (Heavy)**				2
**Treatment with ≥2 Antihypertensives (Hypertensive)**				1
**Atrial Fibrillation (Fibrillation)**				3
**PASP > 35 mm** ** ** **Hg (Pulmonary Hypertension)**				1
**Age ≥ 60 years (Elder)**				1
**Elevated Filling Pressure** **→** **E/e’ ≥ 9 (Filling)**				1
**Probability of HFpEF based on Score**			0–1 → Low, explore other causes	
			2–5 → Intermediate, further testing required	
			6–9 → High, no further testing required	
**HFA-PEFF**				
**Step 1** **→** **Pre-test assessment: HF symptoms, typical risk factors, preserved ejection fraction, no major valve disease or ischemia. Elevated natriuretic peptides support but low levels do not exclude HFpEF**				
**Step 2** **→** **If all the conditions of the previous step are met, then continue with evaluation of three domains: functional, structural and biomarkers**				**Score**
**Functional**	**Major**	septal e′ < 7 cm/sec		2
		lateral e′ < 10 cm/sec		
		mean septal and lateral E/e′ ratio > 15		
		TR Vmax > 2.8 m/sec or systolic pulmonary artery pressure > 35 mmHg		
	**Minor**	mean E/e′ ratio ≥ 9 and ≤14		1
		LV-GLS		
**Structural**	**Major**	In SR LAVI > 34 mL/m^2^ (lean)/29 mL/m^2^ (obese) index to BSA		2
		LAVI > 40 mL/m^2^ for AF index to BSA		
		LVMI > 50 g/m^2.7^(men)/>47 g/m^2.7^(women) index to height + RWT > 0.42		
	**Minor**	LAVI ≥ 29 and ≤ 34 mL/m^2^ for SR		1
		LAVI ≥ 34 and ≤ 40 mL/m^2^ for AF		
		LVMI > 115 g/m^2^ (men)/>95 g/m^2^(women)		
		RWT > 0.42		
		LV wall thickness in the end of diastole ≥ 12 mm		
**Biomarkers**	**Major**	In SR, NTproBNP > 220 pg/mL		2
		In AF, NTproBNP > 660 pg/mL		
		In SR, BNP > 80 pg/mL (lean)/>54 pg/mL (obese)		
		In AF, BNP > 240 pg/mL		
	**Minor**	In SR, NTproBNP ≥ 125 and ≤220 pg/mL		1
		In AF, NTproBNP ≥ 375 and ≤660 pg/mL		
		In SR, BNP ≥ 35 and ≤80 pg/mL		
		In AF, BNP ≥ 105 and ≤240 pg/mL		
**Probability of HFpEF based on Score**			0–1 → Low, explore other causes	
			2–4 → Intermediate, further testing required	
			5–6 → High, no further testing required	
**Step 3** **→ Stress testing, either invasive or non-invasive, is performed for scores in the intermediate range (2–4), to rule out or set the definitive diagnosis of HFpEF**				
**Step 4** **→ U** **ncover the underlying cause of HFpEF:** **A** **dvanced imaging techniques, genetic testing, biopsies, and biochemical laboratory tests**				

AF, atrial fibrillation; BMI, body mass index; H2FPEF, Heavy, Hypertension, Atrial Fibrillation, Pulmonary Hypertension, Elder, and Filling Pressures; HFA-PEFF, Heart Failure Association Pretest Probability of Heart Failure with Preserved Ejection Fraction; HFpEF, heart failure with preserved ejection fraction; LAVI, left atrial volume index; LV-GLS, left ventricular global longitudinal strain; LVMI, left ventricular mass index; PASP, pulmonary artery systolic pressure; RWT, relative wall thickness; SR, sinus rhythm; TR Vmax, tricuspid regurgitation maximum velocity.

## Abbreviations

AF	Atrial fibrillation
BMI	Body mass index
BNP	B-type natriuretic peptide
BSA	Body Surface Area
CCTA	Cardiac computed tomographic angiography
CMR	Cardiac magnetic resonance
H_2_FPEF	Heavy, Hypertension, Atrial Fibrillation, Pulmonary Hypertension, Elder, Filling Pressures
HF	Heart failure
HFA-PEFF	Heart Failure Association Pretest Probability of Heart Failure with Preserved Ejection Fraction
HFpEF	Heart failure with preserved ejection fraction
LA	Left atrium
LAVI	Left atrial volume index
LV	Left ventricle
LVEF	Left ventricular ejection fraction
LVMI	Left ventricular mass index
NP	Natriuretic peptide
NT-proBNP	N-terminal fragment of proBNP
PH	Pulmonary hypertension
RWT	Relative Wall Thickness
TR Vmax	Tricuspid regurgitation maximum velocity
